# Inhibition of DDR1 reduces invasive features of human A375 melanoma, HT29 colon carcinoma and SK-HEP hepatoma cells

**DOI:** 10.1080/19336918.2020.1733892

**Published:** 2020-03-03

**Authors:** Irene Romayor, Iker Badiola, Elvira Olaso

**Affiliations:** Department of Cell Biology and Histology, School of Medicine and Dentistry, University of the Basque Country, Leioa, Spain

**Keywords:** Melanoma, colon carcinoma, hepatocarcinoma, discoidin domain receptor 1, matrix metalloproteinases, collagen receptor, silencing, adhesion, proliferation, migration, invasion

## Abstract

DDR1 is a receptor tyrosine kinases for collagen and an adverse prognostic factor in primary and metastatic tumors.Despite this, DDR1 signaling and its functional consequences in tumor development remain unclear. RT-PCR and Western blot show that A375, colon carcinoma HT29 and liver carcinoma SK-HEP human cell lines express functional DDR1 that phosphorylates in response to collagen type I. Chemical inhibition of DDR1 phosphorylation or DDR1 mRNA silencing reduced AKT and ERK phosphorylation, expression of ICAM1 and VCAM1, Ki67 and secretion of MMP9. DDR1 silenced cells showed reduced adhesion to collagen type I, MMP-dependent invasion, and chemotactic and proliferative responses to collagen type I. Our work indicates an essential role for DDR1 signaling in key prometastatic features of collagen type I in human carcinoma cells.

## Introduction

Discoidin domain receptor 1 (DDR1) belongs to the family of receptor tyrosine kinases for collagen [1–3]. The elevated expression of DDR1 in fast-growing invasive tumors and associated metastases has dramatically increased the researcher´s attention in the pathological implication of DDR1 in cancer [4–6]. Several *in vivo* and *in vitro* studies pin-points DDR1 as a significant promoter of tumor cell invasion. Despite this, the functional consequences of DDR1 signaling are far from being understood. Further use of antitumor therapies based on DDR1 inhibition requires a more in-depth knowledge of cell-specific DDR1 expression and signaling, the mechanisms that activate its signaling, and its functional implication in tumor growth and dissemination. We have previously demonstrated a pro-metastatic role for discoidin domain receptor 2 (DDR2) in A375 melanoma, HT29 colon carcinoma, and SK-HEP hepatocarcinoma cell lines [7]. In this work we utilize two approaches to inhibit DDR1 signaling in those tumor cell lines: chemical inhibition and mRNA silencing. We analyze the effect of DDR1 inhibition in the expression of key signaling mediators for tumor development and analyze the role of DDR1 in pro-invasive cellular functions in response to collagen type I.

## Results

### Human A375, HT29 AND SK-HEP tumor cells express functional DDR1

A375, HT29 and SK-HEP are highly invasive carcinoma cell lines from skin, colon and liver, respectively. We have previously observed that these cell lines are able to metastasize the liver, were tumor development is collagen-dependent by a mechanism partially dependent on DDR2, the other member of the DDR family [7].

**Figure 1.**
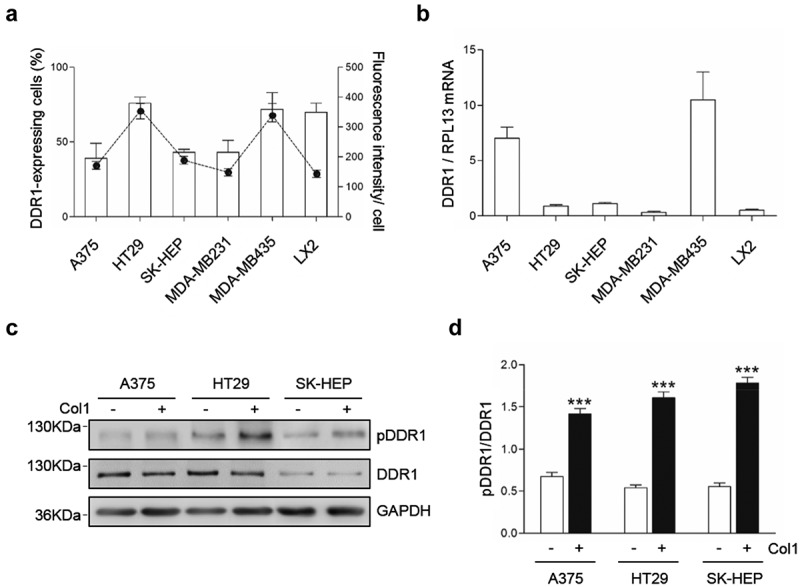
**DDR1 is expressed and phosphorylated in human A375, HT29 and SK-HEP tumor cell lines**. Cells were cultured in serum-free basal media. (a) Some cells were immune-labeled with fluorescent anti-DDR1 antibodies and submitted to flow cytometry analysis on the percentage of cells expressing DDR1 (bars) and the fluorescence intensity of per cell (points). (b) Remaining cells were analyzed for DDR1 mRNA expression by RT-PCR. Tumor cells were cultured in serum-free basal media, in the presence or absence of exogenous Collagen I. (c) Representative Western blot analysis of phosphorylated (pDDR1) and total DDR1 expression in response to exogenous Collagen I. (d) Histogram on computer-assisted semi-quantification of Western Blots for pDDR1 and DDR1 expression in the cells in response to exogenous collagen I from three independent experiments. Data are presented as the means ± standard error, n = 3 (***P < 0.0001).

First, we utilized Flow Cytometry to measure the percentage of cells that expressed the receptor under sub-confluent culture conditions, and the fluorescence intensity per cell, that correlates with receptor density (Figure 1a). MDA-MB231 and MDA-453 were used as a positive control for DDR1 expression in tumor cells, while LX2 cells were used as a positive control for non-tumoral DDR1. DDR1 was detected in all analyzed samples. An average 50% of A375 and SK-HEP cells expressed detectable levels of DDR1, similar to those observed in MDA-MB231 cells, with an average fluorescence intensity of 150AU per DDR1-expressing cell. More than 70% of HT29 cells, MDA-MB453 and LX2 cells showed positive staining, with an average 340AU of fluorescence per DDR1-expressing cells. Next, we studied DDR1 mRNA expression in the three tumor cell lines (Figure 1b). As expected from the flow cytometry data, all cell lines expressed DDR1 mRNA. DDR1 mRNA levels drastically variate between tumor cell lines. A375 cells expressed the highest amount of DDR1 mRNA, similar to that of MDA-MB435, while DDR1 mRNA levels in HT29 and SK-HEP were similar to that of MDA-MB231 and LX2 cells. The discrepancies between mRNA expression and protein expression may indicate that the balance of the processes of production and decay that controls the steady-state levels of DDR1mRNA and/or DDR1 protein is cell type-specific. Finally, we confirm the presence of DDR1 in the lysates of tumor cells cultured in the presence of exogenous collagen I (Figure 1c,d). Western blot against human DDR1 showed a single band of ~125KDa. As previously reported in human hepatoma Huh7 cells [8], tumor DDR1 appeared constitutively phosphorylated in the absence of exogenous collagen. Maximal phosphorylation rates was observed in HT29 cells. Addition of soluble collagen I further phosphorylates DDR1 in the A375, HT29 and SK-HEP cells by an average 2-fold increase compared to basal phosphorylation.

As a whole, this set of experiments demonstrates that DDR1 is expressed and is functional in A375, HT29 and SK-HEP human cancer cells. It also shows that an ample heterogeneity exists among tumor cell lines regarding the percentage of cells expressing DDR1, receptor number per cell and DDR1 mRNA expression.

### Cultured human A375, HT29 and SK-HEP tumor cells produce collagen I

**Figure 2.**
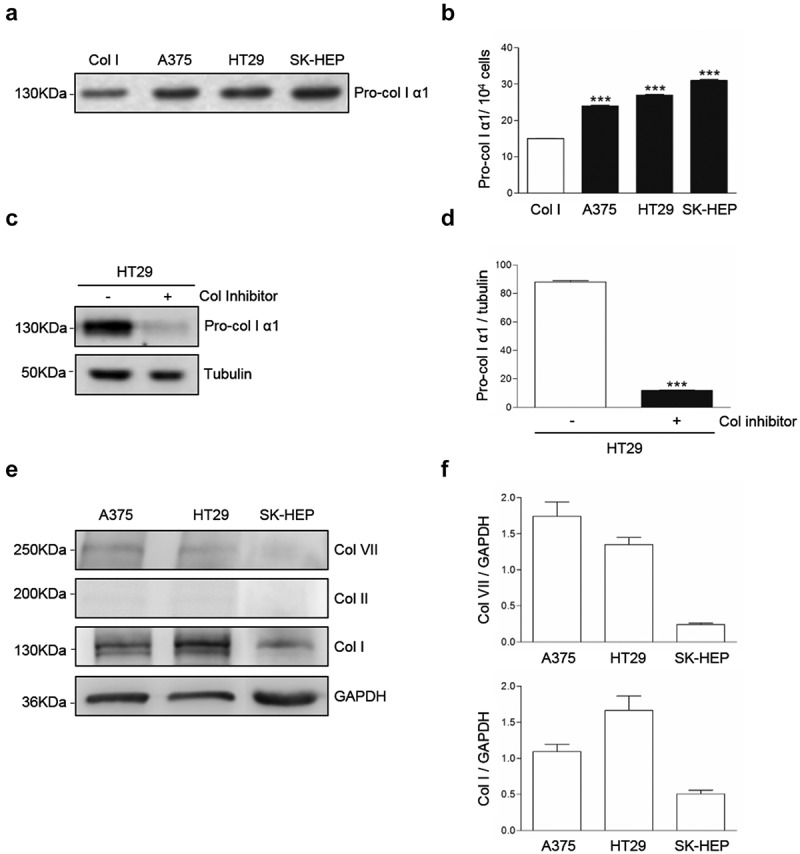
**Collagen type I is a major collagen secreted by A375, HT29 and SK-HEP cells**. Cells were maintained overnight in serum free media. Some cells were treated with the collagen synthesis inhibitor cis-4-hydroxy-L-proline. (a) Representative Western blot analysis of pro-collagen Iα1 expression in the culture supernatants. (b) Computer-assisted semi-quantification of the Western blots for pro-collagen I α1 expression in the culture supernatants. (c) Representative Western blot analysis of pro-collagen I α1 expression in the supernatants of HT29 cells treated or not with a collagen synthesis inhibitor. (d) Computer-assisted semi-quantification of the Western Blots for pro-collagen I α1 expression in HT29 cells treated or not with a collagen synthesis inhibitor. (e) Representative Western blot analysis of the intracellular content of collagens VII, II and I in the cells. (f) Computer-assisted semi-quantification of the Western Blots for intracellular collagens. Data are presented as the means ± standard error, n = 3 (***P < 0.0001).

Western blot analysis of supernatants from A375, HT29 and SK-HEP tumor cells cultures revealed the presence of a band corresponding to pro-collagen I (Figure 2a,b). In order to test if the band corresponded to collagen, HT29 cells were cultured in the presence of the collagen synthesis inhibitor cis4-hidroxy-L-proline under conditions were no other sources of collagen were provided to the cell cultures. As a result, the band was reduced by an 85% (Figure 2c,d), indicating that at least an 85% of the protein detected in the bands corresponded to endogenously produced collagen. Western blot also detected intracellular collagens (Figure 2e,f). Collagen I appears as a major collagen expressed by the tumor cells, compared to expression of collagens II and VII. These results may indicate DDR1 phosphorylation in the tumor cells is triggered by exogenous, but also by endogenous collagen I.

### Chemical blockage of DDR1 phosphorylation reduces downstream phosphorylation of AKT AND ERK, expression of cell-cell adhesion molecules ICAM1 and VCAM1/, secretion of MMP9 and cell proliferation marker

**Figure 3.**
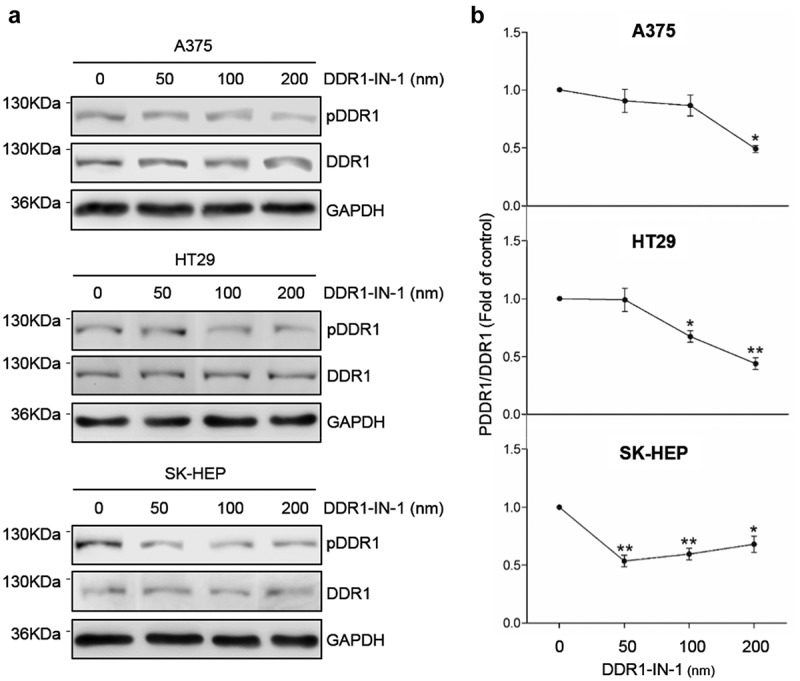
**DDR1-IN-1 blocks of DDR1 phosphorylation in A375, HT29 and SK-HEP cells**. Cells were cultured in serum-free basal media. Some cells received exogenous collagen type I and/or the chemical inhibitor of DDR1 phosphorylation DDR1-IN-1. (a) Representative Western blot analysis of phosphorylated (pDDR1) and total DDR1 expression in cells incubated with collagen I and treated with increasing concentrations of DDR1-IN-1. (b) Computer-assisted semi-quantification of the Western blot for pDDR1 and total DDR1 expression in cells incubated with collagen I and DDR1-IN-1, expressed as a ratio pDDR1/total DDR1. Dotted lines indicate the 50% inhibition of DDR1 phosphorylation (IC50). Data are presented as the means ± standard error, n = 2 (*P < 0.05, **P < 0.001).

Excessive signaling by DDRs has been linked to the progression of various human diseases, including fibrosis, atherosclerosis and cancer, but the signaling mechanism behind it remains unclear. High phosphorylation rates in the tumor cells were obtained by exposure to exogenous collagen type I for two hours (Figure 1c). To reduce DDR1 phosphorylation, tumor cells were treated with the specific DDR1 inhibitor DDR1-IN-1. The IC50 (50% inhibition of DDR1 phosphorylation compared to control) of DDR1-IN-1 has been demonstrated to be sufficient to induce relevant changes in U2OS osteosarcoma human cell line [9]. The IC50 was reached at 200 nM in A375 and HT29 cell lines and at 50 nM in SK-HEP (Figure 3).

**Figure 4.**
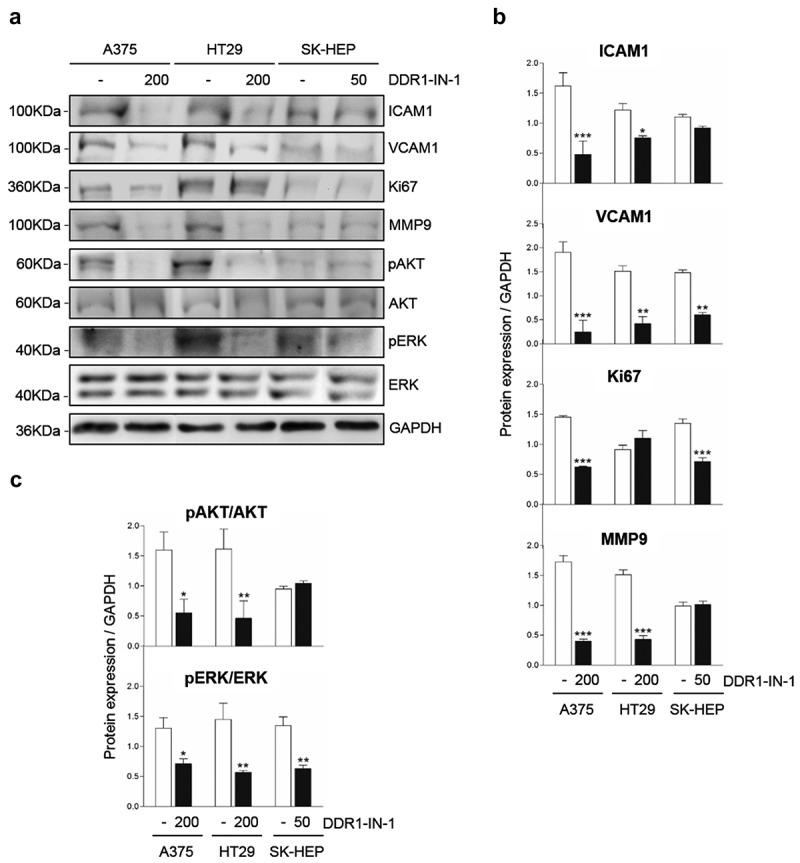
**Chemical blockage of DDR1 phosphorylation modulates receptor downstream signaling in A375, HT29 and SK-HEP cells**. Cells were cultured in serum-free media and received exogenous collagen type I. Some cells also received DDR1-IN-1 in a dose correspondent to the IC50 for each cell line. Remained cells were considered controls. (a) Representative Western blot analysis of ICAM1, VCAM1, Ki67, MMP9, phosphorylated AKT (pAKT), total AKT, phosphorylated ERK (pERK) and total ERK expression in the cells in the presence of exogenous collagen I. (b) Computer-assisted semi-quantification of the Western blots for pDDR1/total DDR1, ICAM1, VCAM1, Ki67 and MMP9 expression. (c) Computer-assisted semi-quantification of the Western blots for pAKT/total AKT and pERK/total ERK expression in the cell (results are presented as the ratio phosphorylated form/total protein). Data are presented as the means ± standard error, n = 3 (*P < 0.05, **P < 0.001, ***P < 0.0001).

Next we used Western Blot to compare the expression levels of several proteins related to tumor cell invasion between cells with high and reduced DDR1 phosphorylation rates (Figure 4). The proteins were selected from a differential genome array data of liver myofibroblast submitted or not to temporary DDR1 silencing (Romayor et al, manuscript in preparation). Reduced expression of adhesion molecules ICAM1 and VCAM1 was observed in the three human cell lines after blocking of DDR1 phosphorylation, but was only significantly altered in A375 and HT29 cells. MMP9 is a key metalloproteinase for extracellular matrix (ECM) degradation during tumor invasion. MMP9 was reduced by ~75% in low DDR1 phosphorylated A375 and SK-HEP cells compared to control ones. DDR1 phosphorylation levels did not affected MMP9 expression in HT29 cells. While inhibition of DDR1 phosphorylation correlates with an ~75% reduction in AKT phosphorylation in A375 and HT29 cells, no significant effect was observed in SK-HEP hepatoma cells. On the other hand, an average ~65% reduction in ERK phosphorylation was found in the three cell lines.

Finally, a role for DDR1 signaling in cell proliferation has been proposed in several tumor cell lines [10]. Expression of the proliferation marker Ki67 [11] was inhibited by ~3-fold by DDR1-IN-1 treatment in A375 and SK-HEP cell lines.

### Silencing of DDR1 expression by small specific interference RNA in the tumor cells reduces phosphorylation of AKT and ERK, secretion of MMP9 and cell proliferation

**Figure 5.**
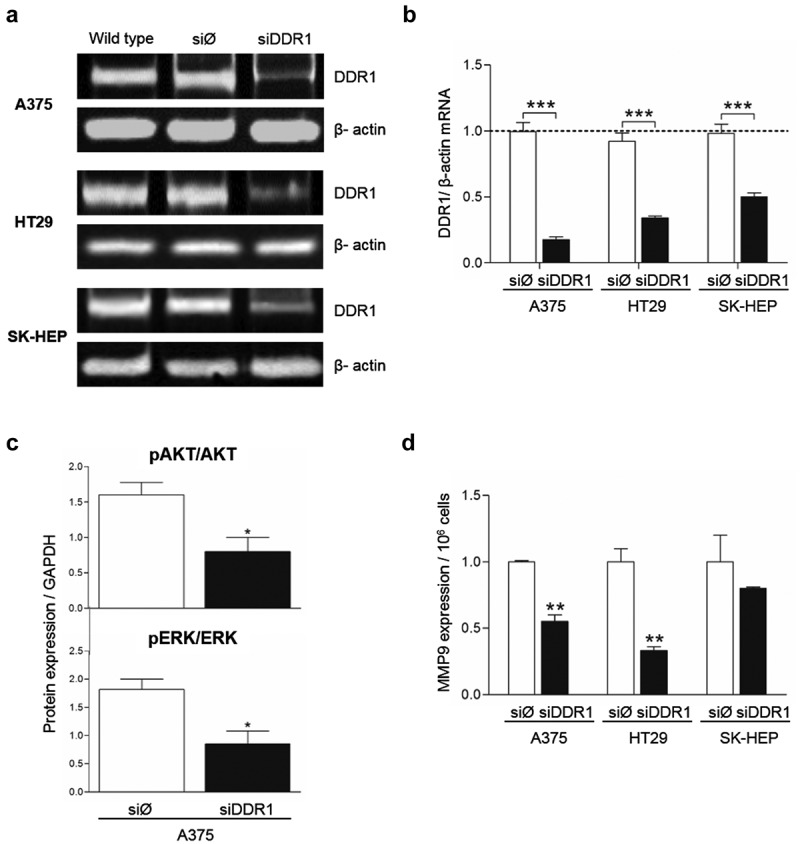
**Silencing of DDR1mRNA in A375, HT29 and SK-HEP cells reduces their expression of MMP9**. Cells were transiently transfected with DDR1 silencing RNA (siDDR1) or with an irrelevant siRNA (siØ). (a) Representative RT-PCR analysis of DDR1 expression in the cells 72 hours after transfection. WT: non-transfected cells. (b) Computer-assisted semi-quantification of DDR1 mRNA expression in the cells after transfections (as fold of wild type values). (c-d) Transfected A375 cells received collagen I. Two hours afterward, supernatants were collected and cells were lysed. (c) ELISA for pAKT, AKT, pERK and ERK in A375 cell lysates. (d) Computer-assisted semi-quantification of MMP9 zymograms of the supernatants. Data are presented as the means ± standard error, n = 3 (*P < 0.05, **P < 0.001, ***P < 0.0001).

**Figure 6.**
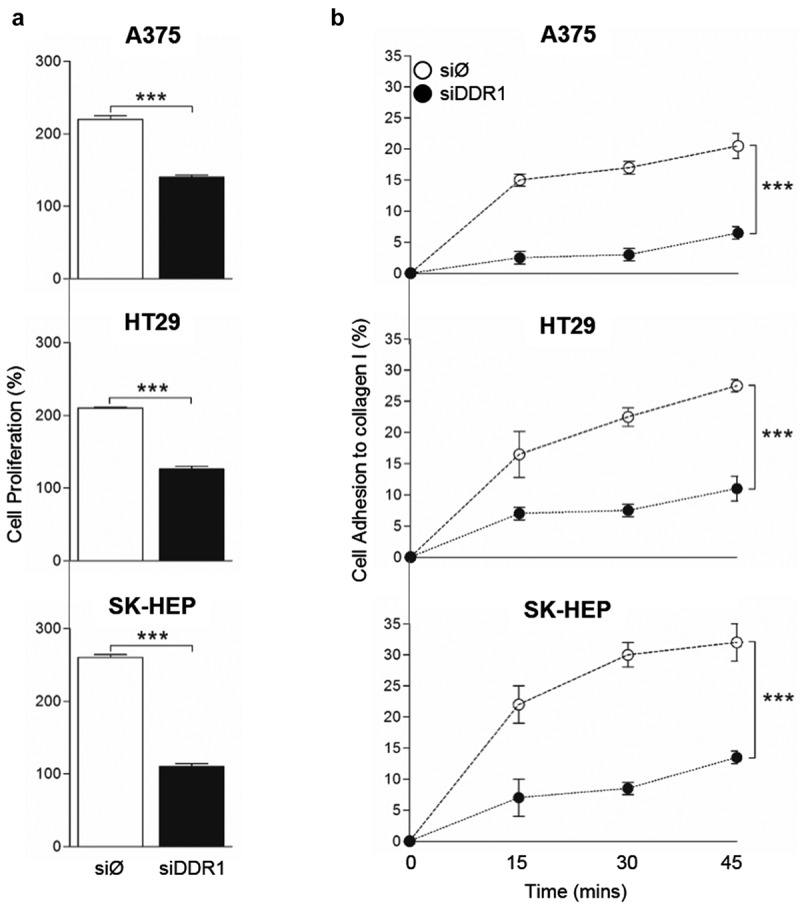
**DDR1 silencing reduces adhesion to collagen type I and proliferation in response to exogenous collagen I in A375, HT29 and SK-HEP cells**. Tumor cells were transiently transfected with DDR1 silencing RNA (siDDR1) or with an irrelevant siRNA (siØ). (a) Proliferation of in response to exogenous collagen type I. (b) Time course of the cell adhesion to collagen I. Data are presented as the means ± standard error, n = 3 (***P < 0.0001).

To strength the results obtained using chemical inhibition of DDR1 phosphorylation, we silenced DDR1 in each tumor cell lines by transient transfection with siRNA against DDR1 (siDDR1), and assayed cell responses in the presence of exogenous collagen. Three days after transfection, silencing efficiency ranged between ~50% in SK-HEP cells and ~80% in A375 cells compared to control cells transfected with scramble siRNA (siØ) (Figure 5a,b). At this time point, DDR1-silenced A375 cells showed a ~ 50% reduction in phospho-AKT and phospho-ERK levels compared to controls (Figure 5c). Also, MMP9 secretion was reduced by 2 to 3-fold in low DDR1 phosphorylated A375 and SK-HEP cells compared to control ones. DDR1 levels did not affected MMP9 expression in HT29 cells. Proliferation rates of DDR1-silenced and control cells were analyzed over a period of 48 hours. Silencing of DDR1 reduced cell proliferation by ~50% (Figure 6a).

Together with the data obtained using DDR1-IN-1 (Figure 4), these results indicate that AKT and ERK phosphorylation rates, MMP9 expression and tumor cell proliferation are modified by DDR1 signaling and/or by DDR1 expression.

### DDR1 silencing reduces the adhesion capacity of human tumor cells to collagen I

The ability of a tumor cell to adhere to collagen rapidly and firmly is a crucial event in tumor cell invasion of the surrounding tissue. Thus, we next performed an assay to measure early strong adhesion of tumor cell to a collagen type I film based on cell ability to remain attached to the surface following cell seeding and media removal after short amounts of time (Figure 6b). An average 15% to 20% of tumor cells treated with the irrelevant siRNA remains adhered to the collagen surface 15 minutes after cell seeding and reach a maximal percentage of 20 to 35% of cells adhered to collagen after 45 minutes. In contrast, silencing of DDR1 reduced very early tumor cell adhesion by an average 75% compared to adhesion of control cells.

### DDR1 silencing reduces migration of human tumor cell lines in response to collagen I

**Figure 7.**
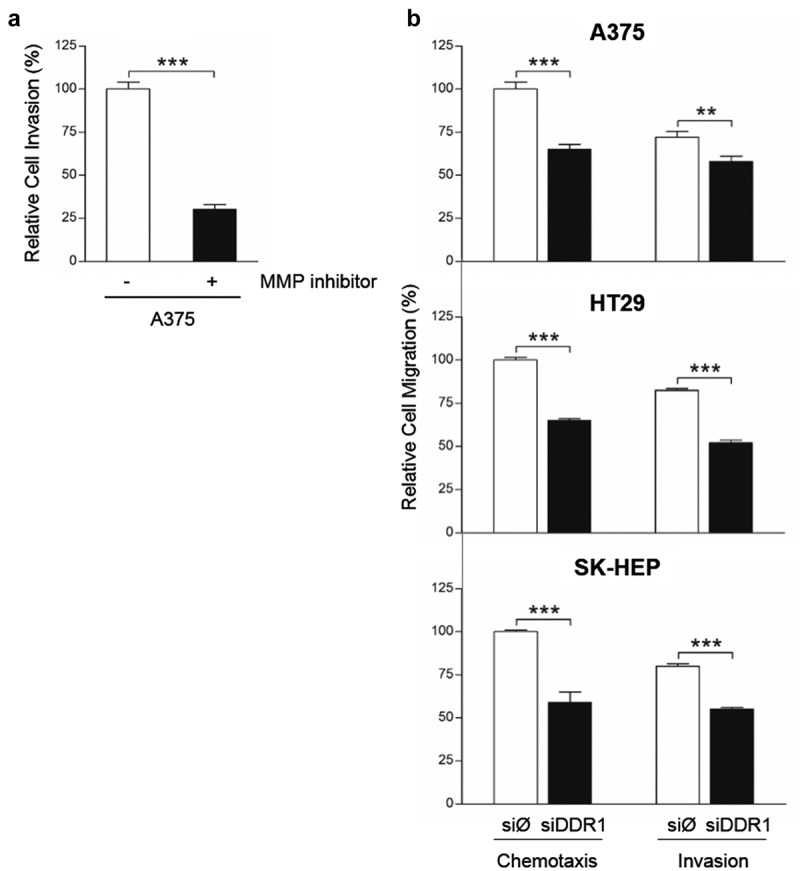
**Silencing of DDR1 mRNA reduces MMP-dependent invasion through collagen I and chemotactic migration in response to exogenous collagen I by A375, HT29 and SK-HEP cells**. (a) A375 cells were treated or not with MMPs synthesis inhibitor, and then analyzed for their ability to invade a collagen I-coated transwell. (b) Chemotactic migration of transiently transfected A375, HT29 and SK-HEP cells in response to exogenous collagen I. Data are presented as the means ± standard error, n = 3 (**P < 0.001, ***P < 0.0001).

In order to test MMPs implication in human tumor cell migration, A375 cells were treated or not with the MMP inhibitor GM6001 and seed in collagen I-coated upper wells of invasion chambers (Figure 7a). Tumor cell invasion was reduced by an average 71% in cells treated with GM6001, compared to untreated cells, indicating that A375 migration was strongly dependent on MMPs synthesis.

Collagen I may serve as chemotactic factor for DDR1-expressing cells [12]. Thus, we carried out chemotaxis assay and tested the ability of siDDR1 cells to invade collagen I-coated wells compared to tumor cells with full expression of DDR1 (Figure 7b). Chemotactic migration toward collagen I was reduced by ~45% in DDR1-silenced cells compared to control ones. Finally, we detected a significant reduction in the invasive capacity of siDDR1 tumor cells, compared to control. Invasion through collagen I was 24 to 41% lower in silenced cells. Thus, the invasion of the collagenous ECM requires both MMPs and DDR1 synthesis by these tumor cells.

## Discussion

In this work, we utilize three human tumor cell lines and two independent approaches to characterize the *in vitro* implication of DDR1 in tumor malignancy through the analysis of tumor responses to collagen I. The first approach utilized a highly selective pharmacological inhibitor for DDR1-dependent signal transduction. The second one is based on DDR1 mRNA silencing. Both approaches demonstrate that DDR1 mediates invasive characteristic such as tumor cell adhesion, proliferation, migration and ECM degradation. An alternative strategy to strength the data could have been the use of a complementary set of siRNA against DDR1.

Blockage of DDR1 expression or phosphorylation in the tumor cells promote changes in a panel of invasion-related proteins, selected from a differential genome array data of liver myofibroblast submitted or not to temporary DDR1 silencing (Romayor et al, manuscript in preparation). We chose ICAM1 and VCAM1, two cell-cell adhesion molecules required for the transmigration step of tumor metastasis [13–15]. VCAM1 expression depends on DDR1 phosphorylation in macrophages [16].

Adhesion of SK-HEP-1 cells to the basal membrane and ECM is mediated by ICAM1 activation [17]. In this work, we established a direct correlation between DDR1 phosphorylation and tumor cell expression of ICAM1 and VCAM1.

We also studied the role of DDR1 in the expression of MMP9, a key regulator of ECM remodeling implicated in tumor invasion through the collagenous microenvironment and in tumor growth [18,19]. DDR1 mediates MMP9 induced by native type IV collagen in MDA-MB-231 breast cancer cells [20]. Our work demonstrated that collagen type I also induces DDR1 mediated synthesis of MMP9 in tumor cells. Finally, we assayed two major downstream effectors of tyrosine kinase receptors, AKT and ERK [21]. Our data indicates that DDR1 signaling is a major source of AKT and ERK phosphorylation in tumor cells.

Tumor colonization progress as tumor cells migrates in response to chemotactic factors matrix metalloproteinases provided by the tumor microenvironment to degrade the natural barriers such as the basal membrane or ECM [22]. In this work, we show that chemical inhibition of MMPs strongly reduces A375 tumor cell invasive migration and that blockage of DDR1 expression reduces tumor cell synthesis of MMP9, while it also reduces proliferation and migration. MMPs facilitate tumor cell invasion and metastasis. As an MMP superfamily member, MMP2 and 9 can degrade the proteins of the ECM of cancer cells, and they can also promote metastatic tumor cells to penetrate the basal membrane, leading to invasion and metastasis.

During tumor dissemination, circulating tumor cells must adhere to and confront a collagenous stroma endowed with pro-adhesive but anti-proliferative and pro-apoptotic properties [23,24]. DDR1 promotes adhesion to collagen of a variety of malignant cells, including pituitary adenoma, glioma, and hepatoma cells [24]. Opposed results were obtained by Valencia and cols using H460 lung carcinoma cells, indication that adhesion is a complex process as a result of multiple DDR1-dependent and independent pathways. Our results indicate that phosphorylated DDR1 mediates the initial adhesion of A375, HT29, and SK-HEP to type I collagen. DDR1 mediation of cell adhesion to collagen gene may also be independent of DDR1 kinase domains, as recently demonstrated [25]. Finally, DDR1 may also affect the ability of other collagen receptors to promote cell adhesion, although it may not be through DDR1-DDR2 interactions [25].

Once adhered, tumor cells must proliferate. In this work, we found that DDR1 mediates proliferation in a collagenous culture setting of the three tested tumor cell lines. Furthermore, we found a direct correlation between DDR1 silencing and the levels of phosphorylated AKT and ERK. AKT and ERK signaling are two pro-survival molecules in tumor cells under genotoxic stress, but their role in the proliferation or survival of tumor cells under favorable conditions remains unknown. While several adaptor proteins have been identified that bind DDR1 in response to collagen stimulation, the exact DDR1 signaling pathway elicited to promote a cell function may be significantly cell-specific. Furthermore, DDR1 inhibitors may reduce DDR1 signals through only one of them. For example, DDR1 promotes cell migration through ERK signaling in lymphocytes [26], but not in human lung fibroblast [27].

In this work, we address whether DDR1 phosphorylation is stromal-dependent or may also depend on endogenous collagen secretion. The second hypothesis arises from the fact that the three carcinoma cells secrete collagen type I to the medium. While this result is pending of further analysis by mass spectrometry, it is tempting to say that endogenous collagen produces continuous DDR1 phosphorylation and signaling in these tumor cells, which may indicate a potential positive loop between DDR1 signaling and collagen type I. Alternatively, other collagen receptors may result altered in response to DDR1 silencing. Integrins are altered in DDR1 knock out HEK 293 cells [10]. Also, we have previously demonstrated that A375, HT29, and SK-HEP cells express functional DDR2, the other member of the DDR family [7]. Finally, DDR1 and DDR2 physical interaction lead to signaling interconnection [25]. Whether the same mechanism acts in tumor cells remains unknown.

The *in vitro* results presented in this work open the door for future *in vivo* and *in vitro* experiments to confirm its functional relevance. For example, it would be relevant to analyze tumor responses in conditions that more closely resembles the in vivo situation such as culture in 3D collagen gels.

## Material and methods

### Cell lines

The human malignant melanoma A375, colon carcinoma HT29, hepatocarcinoma SK-HEP and breast cancer MDA-MB231 and MDA-MB435 were obtained from the American Type Culture Collection (ATCC, LGC Standards SLU). Human LX2 cell line is derived from hepatic stellate cells [28] and were a kind gift of Dr. S. Friedman (ICAH School at Mount Sinai School of Medicine, New York). A375, HT29 and SK-HEP were cultured in RPMI-1640 medium; MDA-MB231 and MDA-MB435 in Leibovich medium and LX2 cells were maintained in DMEM medium; all purchased from Thermo Fisher Scientific (MA, USA). Media were supplemented with 10% fetal bovine serum (FBS), penicillin (100 U/ml), streptomycin (100 µg/ml) and amphotericin B (0.25 µg/ml), all purchased from Thermo Fisher Scientific. Human cell lines were discarded after ten passages and substituted by new batches. In some experiments, human tumor cells were maintained in serum-free media for up to 3 hours in the absence or presence of 10 nM rat tail collagen type I (native form, Thermo Fisher Scientific), 1 mM collagen synthesis inhibitor cis-4-hydroxy-L-proline (cis-H-pro; Sigma-Aldrich, MI, USA) or DDR1-IN-1 (Tocris Bioscience, United Kingdom), a highly selective pharmacological probe for DDR1-dependent signal transduction [9].

### RT-PCR

Total RNA was extracted using Norgen FFPE RNA Purification kit (Norgen Biotek Corp., Canada) according to the manufacturer´s instructions. After RNA concentration and quality were assessed by NanoDrop spectrophotometer ND-1000 (Thermo Fisher Scientific). 1 µg of RNA was reverse transcribed into cDNA with iScript^TM^ Reverse Transcription Supermix (BIO-RAD, CA, USA). Then, quantification of cDNA template was performed with RT-PCR using iTaq^TM^ Universal SYBR® Green Supermix (BIO-RAD) in ABI 7900HT (Thermo Fisher Scientific). PCR primers (Thermo Fisher Scientific) were as follows: DDR1 F, 5ʹ-CCCTGGTTACTCTTCAGCGAAAT-3 and R, 5ʹ-AACACCCTCCGTTCAGCCTT-3ʹ. The cycle number was optimized to ensure that the amplification was in the linear range. PCR products were visualized on a 2% agarose gel with ethidium bromide, and the resultant bands were quantified with FIJI–ImageJ.

### Flow cytometry

Human tumor cells were dissociated with nonenzymatic cell dissociation solution (Sigma-Aldrich) and resuspended in PBS containing 1% BSA. The cells were incubated for 1 hour at room temperature with anti-DDR1 (GeneTex, CA, USA) followed by allophycocyanin-conjugated secondary antibody (Thermo Fisher Scientific). Samples labeled only with the secondary antibody were used as negative controls. After three washes as above, the cells were resus- pended in 2% formaldehyde in PBS. Cell viability was analyzed by propidium iodide (Sigma-Aldrich) staining. Finally, cells were analyzed by flow cytometry (FACScalibur, BD Biosciences, USA) at 488 nm. Data acquisition was performed on CellQuest Pro Software at 1024 channel resolution and the fluorescence logarithmic amplification analyses were performed using Paint-A-Gate Pro Software (BD Biosciences).

### Western blot

Human tumor cells were washed twice with cold 1X PBS before adding 1X Laemmli sample buffer (BIO-RAD) with 1% beta-mercaptoethanol (Sigma-Aldrich) (100 *µ*l/10^6^ cells). Protein quantification was performed using trichloroacetic acid (TCA) precipitation (Fluka Biochemika, ‎Switzerland). 20 µg of cell protein lysates were used. Then, samples electrophoresis was performed in 10% SDS PAGE gels and was run 1 hour 30 minutes at 90 mV. Proteins were transferred onto a nitrocellulose membrane in wet conditions at 385 mA for 3 hours 30 minutes on ice.

Membranes were incubated overnight at 4°C with rabbit anti-pDDR1 (1:500; St John´s Laboratory, United Kingdom), rabbit anti-DDR1 N-terminal (1:1000; GeneTex), mouse anti-GAPDH (1:1000; BIO-RAD), rat anti–ICAM1 (1:1000; Thermo Fisher Scientific), rat anti–VCAM1 (1:1000; R&D Systems, MI, USA), mouse anti-Ki67 (1:1000, Abcam, United Kingdom), rabbit anti-MMP9 (1:1000, Abcam), Rabbit anti-pAKT (1:500 St John´s Laboratory), rabbit anti-AKT (1:100; St John´s Laboratory), mouse anti-pERK (1:500; St John´s Laboratory, rabbit anti-ERK (1:1000; Cell Signaling Technology, MA, USA) and mouse anti-GAPDH (1:1000). One hour incubation with specific biotinylated secondary antibodies goat anti-rabbit (1:1500), rabbit anti-mouse (1:2000) and goat anti-rat (1: 2000), all from Thermo Fisher Scientific, was followed by a thirty minutes incubation with streptavidin-HRP conjugated (1:500) or ultra-Sensitive ABC Peroxidase Staining Kit (Thermo Fisher Scientific).In order to detect intracellular collagen, cells were maintained in serum-free media overnight and processed as described above. Then, membranes were incubated with mouse anti-Col VII α1 (1:750; Sigma-Aldrich), rabbit anti-Col II α1 (1:500; GeneTex), rabbit anti- Col I α1 (1:1000; Abcam) and mouse anti-GAPDH (1:1000); followed by one hour incubation with specific biotinylated secondary antibodies, as explained above.

Collagen content was also detected in the supernatants of cells cultured for 24 hours in serum-free media. Supernatants aliquots from an identical number of tumor cells were centrifuged to eliminate cell debris, submitted to SDS/electrophoresis and proteins transferred to membranes as described above. Membranes were probed with a polyclonal antibody against the carboxyl terminus of human collagen I α1 (Santa Cruz Biotechnology, TX, USA), followed by incubation with anti-rabbit IgG antibodies.

Bands were visualized using Super Signal Femto Substrate kit (Pierce Biotechnology, MA, USA) or using Luminata^TM^ Crescendo Western HRP Substrate (Millipore Corporation, MA, USA). All bands were imaged with SynGene chemiluminescence box, and FIJI–ImageJ was used to semi-quantify protein expression.

*In vitro inhibition of DDR1 phosphorylation*. Cells were incubated one hour with increasing concentrations of DDR1-IN-1 in serum-free media. Then, media was changed and cells were incubated in serum-free media plus 10 nM collagen I and DDR1-IN-1 for 2 hours. Cells were washed twice with cold 1X PBS and processed for Western blot.

### Cell transfection

RNA against DDR1 (siDDR1) was designed against the following sequence: AAU-GUG-CGU-AAG-GGA-CAC-CCU, and used it conjugated or not with Alexa 488 (Quiagen NV, Germany). The non-targeting control sequence (siØ) was designed against AAU-UCU-CCG-AAC-GUG-UCA-CGU, and we used it conjugated or not with FITC (Quiagen). Lipofectamine vesicles (Thermo Fisher Scientific) were incubated with siDDR1 or siØ, and then added to sub-confluent human tumor cell cultures. After 72 hours, transfection efficiency was measured by counting the number of cells transfected with siDDR1 or siØ conjugated with their respective fluorochrome under fluorescence microscopy. In parallel, silencing efficiency of non-conjugated siRNAs was measured by RT-PCR.

### Zymographic detection of metalloproteinases (MMPs)

MMPs expression was assayed by gelatin zymography as previously described [29]. In brief, human tumor cells were cultured in serum-free media for 24 hours and incubated overnight with or without 10 nM collagen type I. Supernatant aliquots from an identical number of tumor cells were centrifuged to eliminate cell debris and submitted to 10% gelatin zymography. Results were semi-quantified using FIJI–ImageJ.

*ELISA assay*. Cells were cultured in basal media with or without exogenous 10 nM collagen type I for two hours. Once fixed, cells were incubated with anti-AKT, anti-ERK and anti-phosphoprotein antibodies, followed by HRP-conjugated secondary antibody and the correspondent substrate. The signal was measured as absorbance at 650 nm in an ELISA reader (Thermo Fisher Scientific).

### Cell adhesion to collagen type I

This method is based on cell ability to remain attached to the surface following cell seeding and media removal after short amounts of time. First, plates were pre-coated with 1% collagen type I for 1 hour. Then, human tumor cells were incubated with the fluorescent probe CFSE (BD Biosciencies) for 30 minutes and seeded onto the coated dishes. Fluorescent emission after 0, 15, 30 and 45 minutes was measured at 485 nm and 530 nm.

### Proliferation assay

Human tumor cells were cultured in serum-free media, with or without 10 nM collagen type I. After 24 hours, MTT assay (Abcam) was performed according to the manufacturer´s instructions. Proliferation index was calculated as the percentage of the initial cell concentration in the cultures.

*Invasion and migration assay*s were carried out in modified Boyden chambers as previously described [30], with some minor modifications. Human tumor cells were placed on top of 0.8 µm-pore diameter inserts (Greiner Bio-one, Austria) in serum-free media. For invasion analyses, inserts were pre-coated with 1% collagen type I and serum-containing media was placed in the lower compartment as chemoattractant. For chemotactic migration analyses, inserts remained uncoated and the lower compartment of the chamber contained 10 nM collagen type I in basal serum-free media. In some experiments, A375 tumor cells invasion was performed in the presence of 1 µM GM6001 (Thermo Fisher Scientific), a synthetic hydroxyamate inhibitor of MMPs synthesis as described [30]. Cells were allowed to migrate for 4 hours, and counted in six 20x-fileds per insert using FIJI–ImageJ.

### Statistical analysis

Differences in gene and protein expression levels were analyzed by Student’s t test. Between-group differences in adhesion, proliferation, invasion and migration were analyzed by Anova test. The experiments were performed in triplicate. The criterion for significance was P ≤ 0.05 for all comparisons. Data are expressed as the mean ± SD.
